# lncRNA DARS-AS1 Promoted Osteosarcoma Progression through Regulating miR-532-3p/CCR7

**DOI:** 10.1155/2022/4660217

**Published:** 2022-04-05

**Authors:** Yan Xue, Hongmiao Liu, Guangchen Nie, Xiaoping Ren

**Affiliations:** ^1^Hand and Microsurgery Center, The Second Affiliated Hospital of Harbin Medical University, Harbin 150081, China; ^2^Department of Orthopaedics, The fifth hospital of Harbin, Harbin Heilongjiang 150040, China; ^3^Department of Pathology, The general hospital of Heilongjiang Farms & Land reclamation administration, Harbin, Heilongjiang 150086, China; ^4^State Province Key Laboratories of Biomedicine Pharmaceutics, Harbin Medical University, Harbin, Heilongjiang 150086, China; ^5^Heilongjiang Medical Science Institute, Harbin Medical University, Harbin, Heilongjiang 150086, China

## Abstract

**Background:**

lncRNAs have been indicated to involve in cell invasion, proliferation, and metastasis. However, function of DARS-AS1 in osteosarcoma remains poorly explored.

**Methods:**

DARS-AS1 and miR-532-3p level were measured using qRT-PCR. CCK-8 assay and cell invasion assay were done to study cell functions. Luciferase reporter assay was performed to study the mechanism about DARS-AS1 and miR-532-3p.

**Results:**

We firstly showed that DARS-AS1 expression is upregulated in 73.5% (25/34) of cases with osteosarcoma. Moreover, DARS-AS1 expression is overexpressed in osteosarcoma specimens than in nontumor samples. The DARS-AS1 is overexpressed in the osteosarcoma cell lines (Saos-2, SOSP-9607, U2OS, and MG-63) compared to hFOB. Overexpression of DARS-AS1 promotes cell growth and invasion in MG-63 osteosarcoma cell. DARS-AS1 plays as one sponge for miR-532-3p in osteosarcoma cell, and miR-532-3p overexpression inhibits luciferase activity of DARS-AS1-WT, not DARS-AS1-MUT in MG-63 cell. Ectopic expression of DARS-AS1 inhibits miR-532-3p expression in MG-63 cell. Furthermore, miR-532-3p expression is downregulated in osteosarcoma specimens compared to in paired nontumor samples. MiR-532-3p expression is downregulated in osteosarcoma cell lines compared to hFOB. MiR-532-3p expression is negatively associated with DARS-AS1 expression in osteosarcoma specimens. miR-532-3p directly regulates CCR7 expression in osteosarcoma cell. Elevated DARS-AS1 expression enhances cell growth and invasion via regulating CCR7.

**Conclusions:**

These data firstly suggested that DARS-AS1 exerted as one oncogene in osteosarcoma partly via regulating miR-532-3p/CCR7.

## 1. Background

Osteosarcoma is a primary bone malignancy that influences growing bones of adolescents and children and is interrelated with high morbidity [1–3]. The development of several therapeutic methods for osteosarcoma such as radiotherapy, multiagent chemotherapy, and precise tumor excision has ameliorated the prognosis of osteosarcoma [4–6]. However, the five-year surgical rate of these patients diagnosed with advanced stage is still discontent [7–9]. Thus, it is urgent to find novel biomarkers and treatment targets for osteosarcoma cases.

Long noncoding RNAs (lncRNAs) are one type of the noncoding RNAs (ncRNAs) with length exceed that of 200 nucleotides and can modulate gene expression in posttranscriptional or transcriptional level [10–13]. Recent data have suggested that lncRNAs play crucial roles in a lot of cellular functions including differentiation, invasion, proliferation, migration, development, apoptosis, and metastasis [14–18]. Increasing evidences have revealed that lncRNAs are dysregulated in various cancers such as lung carcinoma, bladder cancer, gastric carcinoma, hepatocellular carcinoma, and also osteosarcoma [19–24]. Studies revealed that DARS-AS1 exerted oncogenic roles in human tumors such as thyroid cancer, myeloma, lung cancer, and ovarian cancer [25–28]. For example, Zheng et al. [27] revealed that DARS-AS1 level was increased in thyroid tumor specimens and was associated with poor prognosis, distant metastasis, and pathological stage, and DARS-AS1 facilitated thyroid tumor cell migration and proliferation via regulating miR-129. Liu et al. [28] demonstrated that DARS-AS1 induced nonsmall cell lung tumor progression through modulating miR-532-3p. Yan [26] showed that DARS-AS1 was upregulated via HIF-1 in myeloma. DARS-AS1 induced myeloma cell tumorigenesis and survival via binding RBM39. Huang et al. [25] showed that expression of DARS-AS1 was upregulated in ovarian tumor specimens and silenced DARS-AS1 expression suppressed cell invasion, migration, and proliferation. However, its function in osteosarcoma remains poorly explored.

We firstly revealed that the DARS-AS1 expression was upregulated in osteosarcoma specimens and cell lines. DARS-AS1 promoted cell growth and invasion in MG-63 osteosarcoma cell.

## 2. Experimental Materials and Methods

### 2.1. Clinical Specimens

Thirty pairs of osteosarcoma tissues and pair no-tumor samples were collected from the Second Affiliated Hospital of Harbin Medical University and immediately stored in the liquid nitrogen.

### 2.2. Cell Culture and Transfect

Four osteosarcoma cell lines (Saos-2, MG-63, SOSP-9607, and U2OS) and a normal osteoblast line (hFOB) were obtained from the American Type Culture Collection (ATCC, USA). These cell lines were plated in the DMEM medium supplemented with FBS, penicillin, and streptomycin. siRNA-control and siRNA-CCR7, MiR-532-3p mimic and scramble, pcDNA-DARS-AS1, siRNA-DARS-AS1, and pcDNA-control and siRNA-control plasmids were collected from the Ribobio (Guangzhou, China). Cell transfection was conducted by using Lipofectamine2000 (Invitrogen, CA, USA) according to the manufacturer's protocol. The sequences of siCCR7: 5′-GCGUC AACCC UUUCU UGUATT-3′ and 3′-UACAA GAAAG GGUUG ACGCAG-5′; miR-532-3p mimic: 5′-CCUCCCACACCCAAGGCUUGCA-3′.

### 2.3. RNA Extraction and qRT-PCR

Total cellular and tissue RNA was extracted by using TRIzol kit (Invitrogen, USA) following manufacturer's instruction. Complementary DNAs (cDNAs) were composed, and qRT-PCR analysis was done using SYBR Green (Bio-Rad, Berkeley, CA) on the real-time PCR ABI7500 instrument. The expression of lncRNA and mRNA was compared to GAPDH using the 2^-DDCT^ way. qRT–PCR primers were amplified as follows: GAPDH: forward 5′-AGGTCCACCACTGACACGTT-3′, reverse, 5′-GCCTCAAGATCATCAGCAAT-3′; miR-532-3p: forward 5′-CCUCCCACACCCAAGGCUUGCA-3′, reverse, 5′-CAAGCCUUGGGUGUGGGAGGUU-3′; DARS-AS1: forward 5′-AGCCAAGGACTGGTCTCTTTT-3′, reverse, 5′-CTGTACTGGTGGGAAGAGCC-3′; and miR-532-3p: forward 5′-CGTTT CCAAC TGTATG-3′, reverse, 5′-CAACGGCGGATGGCC-3′. The following conditions of qRT-PCR were noted: 40 seconds at the 95°C, 45 cycles for 12 seconds at 95°C, and 60°C for 40 seconds.

### 2.4. Cell Proliferation and Invasion Assay

For cell proliferation, cell was kept into the 96-well dish at 5 × 10^3^ cells/well. The cell growth rate was analyzed using CCK-8 (DOJINDO, Japan) following manufacturer's protocol at the different time points. The absorbance at 450 nM was determined on the microtiter reader. For cell invasion, Bio-Coat Matrigel chambers (BD Biosciences, Germany) was used. For cell invasion assay, cells were seeded on the top chamber (Matrigel coated filter) in the serum-free medium, and FBS (10%) was conducted as a chemoattractant. After incubation for 48 hours, the cell that invaded to lower side was fixed and counted.

### 2.5. Luciferase Reporter Assay

Cell was cotransfected pMIR vector containing the diverse mutant or wild type DARS-AS1 and mutant or wild type CCR7, along with pRL-TK control plasmid and miR-532-3p mimic or scramble control by using Lipofectamine2000 (Invitrogen, USA). After 2 days, cell was harvested and then analyzed with the Dual Luciferase Assay kit (Promega, USA) following manufacturer's protocol.

### 2.6. Statistical Analysis

Data are indicated as means + SD (Standard Deviation) based on 3 independent experiments and determined by using SPSS version 12.0 software (SPSS, Chicago, USA). Statistical significance was regarded as *P* value < 0.05. The significant difference was analyzed by one-way analysis of variance or Student's *t* tests.

## 3. Results

### 3.1. DARS-AS1 Was Highly Expressed in Osteosarcoma Cells and Specimens

**Figure 1:**
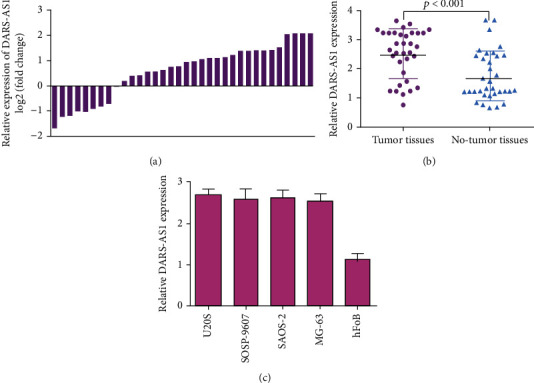
DARS-AS1 was highly expressed in osteosarcoma cells and specimens. (a) The DARS-AS1 expression in osteosarcoma specimens and pair no-tumor samples was analyzed with qRT-PCR analysis. (b) DARS-AS1 expression was upregulated in the osteosarcoma specimens compared to in the pair no-tumor samples. (c) The DARS-AS1 expression was overexpressed in osteosarcoma cell lines (U2OS, SOSP-9607, Saos-2, and MG-63) than in one normal osteoblast line (hFOB).

DARS-AS1 expression in osteosarcoma specimens and paired nontumor samples was analyzed with qRT-PCR analysis. As presented in Figure 1(a), DARS-AS1 expression was upregulated in 73.5% (25/34) of cases with osteosarcoma. Moreover, DARS-AS1 expression was higher in osteosarcoma specimens than in paired nontumor samples (Figure 1(b)). DARS-AS1 expression was overexpressed in osteosarcoma cell lines (U2OS, SOSP-9607, Saos-2, MG-63, and HOS) than in one normal osteoblast line (hFOB) (Figure 1(c)).

### 3.2. miR-532-3p Expression Was Decreased in Osteosarcoma Cells and Specimens

**Figure 2:**
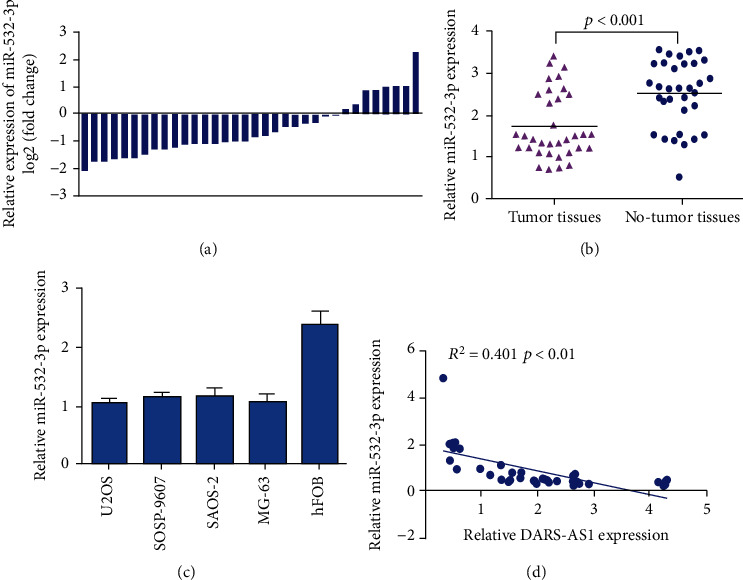
miR-532-3p was decreased in osteosarcoma cells and specimens. (a) The miR-532-3p expression in osteosarcoma specimens and pair no-tumor samples was analyzed with qRT-PCR method. (b) miR-532-3p expression was lower in the osteosarcoma specimens than in the pair no-tumor samples. (c) The miR-532-3p expression was downregulated in osteosarcoma cell lines (U2OS, SOSP-9607, Saos-2, and MG-63) compared to in one normal osteoblast line (hFOB). (d) Pearson's correlation assay indicated that miR-532-3p expression was negatively associated with DARS-AS1 expression in osteosarcoma specimens.

miR-532-3p expression in osteosarcoma specimens and paired nontumor samples was analyzed with qRT-PCR method. As presented in Figure 2(a), miR-532-3p expression was downregulated in 76.5% (26/34) of cases with osteosarcoma. Moreover, miR-532-3p expression was lower in the osteosarcoma specimens than in the paired nontumor samples (Figure 2(b)). miR-532-3p expression was decreased in osteosarcoma cell lines (U2OS, SOSP-9607, Saos-2, MG-63, and HOS) than in hFOB (Figure 2(c)). Furthermore, Pearson's correlation assay indicated that miR-532-3p expression was negatively associated with DARS-AS1 expression in osteosarcoma specimens (Figure 2(d)).

### 3.3. DARS-AS1 Played as a Sponge for miR-532-3p in Osteosarcoma Cell

**Figure 3:**
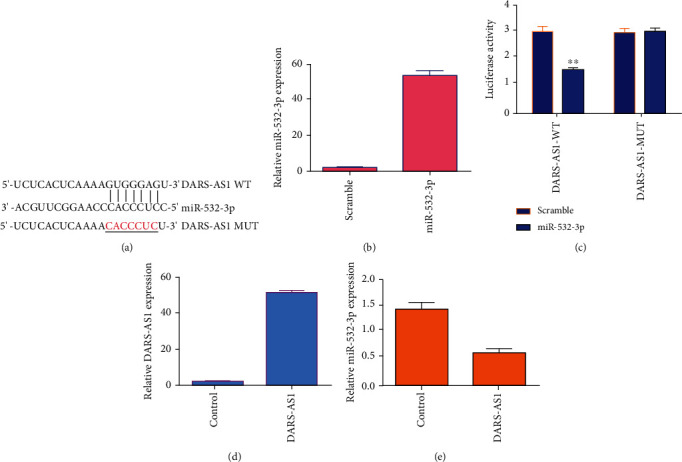
DARS-AS1 played as a sponge for miR-532-3p in osteosarcoma cell. (a) DARS-AS1 has potential binding sites of miR-532-3p. (b) The expression of miR-532-3p was significantly upregulated in the MG-63 osteosarcoma cell after treated with miR-532-3p mimics. (c) Overexpression of miR-532-3p decreased luciferase activity of DARS-AS1-WT, not DARS-AS1-MUT in the MG-63 cell. (d) The expression of DARS-AS1 was significantly upregulated in the MG-63 osteosarcoma cell after treated with pcDNA-DARS-AS1 plasmid. (e) Ectopic expression of DARS-AS1 inhibited the miR-532-3p level in the MG-63 cell. ^∗∗^*p* < 0.01.

To study the relationship between DARS-AS1 and miR-532-3p, we observed that DARS-AS1 has potential binding sites of miR-532-3p (Figure 3(a)). The expression of miR-532-3p was significantly upregulated in MG-63 osteosarcoma cell after treated with miR-532-3p mimics (Figure 3(b)). Then, we carried out luciferase analysis to indicate that overexpression of miR-532-3p decreased luciferase activity of DARS-AS1-WT, not DARS-AS1-MUT in the MG-63 cell (Figure 3(c)). The expression of DARS-AS1 was significantly upregulated in the MG-63 osteosarcoma cell after treated with pcDNA-DARS-AS1 plasmid (Figure 3(d)). Ectopic expression of DARS-AS1 inhibited miR-532-3p level in MG-63 cell (Figure 3(e)).

### 3.4. miR-532-3p Directly Regulated CCR7 Expression in Osteosarcoma Cell

**Figure 4:**
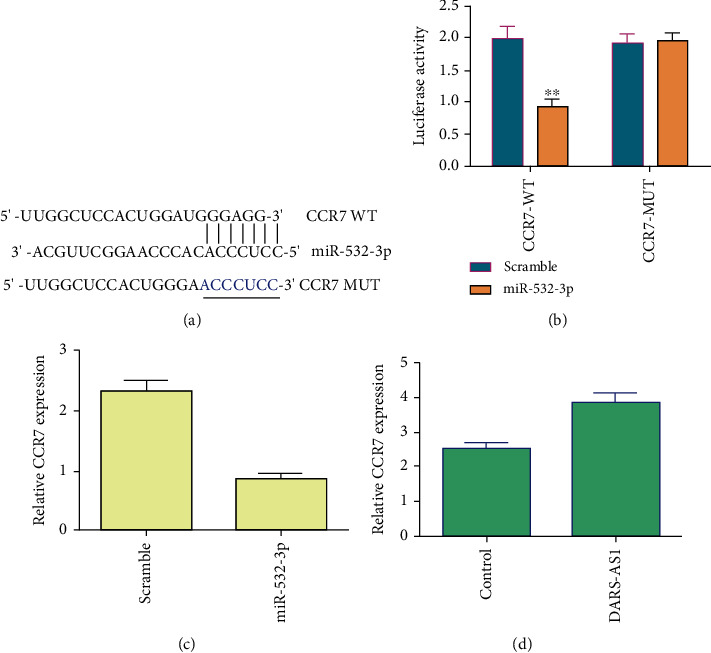
miR-532-3p directly regulated CCR7 expression in osteosarcoma cell. (a) CCR7 has potential binding sites of miR-532-3p. (b) Overexpression of miR-532-3p suppressed luciferase activity of CCR7-WT, not CCR7-MUT in the MG-63 cell. (c) Elevated expression of miR-532-3p inhibited the CCR7 expression in the MG-63 cell. (d) Ectopic expression of DARS-AS1 promoted CCR7 expression in the MG-63 cell. ^∗∗^*p* < 0.01.

To study the relationship between CCR7 and miR-532-3p, we observed that CCR7 has potential binding sites of miR-532-3p (Figure 4(a)). Then, we carried out luciferase analysis to show that overexpression of miR-532-3p suppressed luciferase activity of CCR7-WT, not CCR7-MUT in the MG-63 cell (Figure 4(b)). Elevated expression of miR-532-3p inhibited the CCR7 expression in the MG-63 cell (Figure 4(c)). Moreover, ectopic expression of DARS-AS1 promoted CCR7 expression in the MG-63 cell (Figure 4(d)).

### 3.5. DARS-AS1 Promoted Cell Growth and Invasion in MG-63 Osteosarcoma Cell

**Figure 5:**
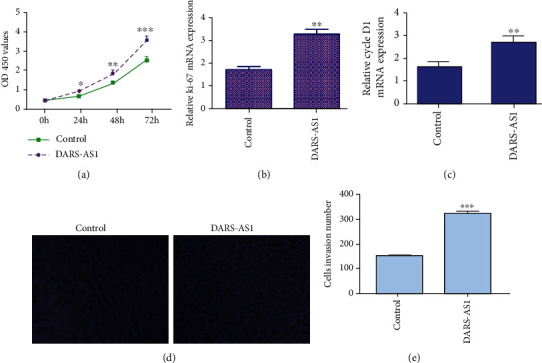
DARS-AS1 promoted cell growth and invasion in MG-63 osteosarcoma cell. (a) CCK-8 assay results indicated that overexpression of DARS-AS1 enhanced cell proliferation both in the MG-63 cells. (b) Ectopic expression of DARS-AS1 increased ki-67 expression both in the MG-63 cells. (c) Elevated expression of DARS-AS1 promoted cyclin D1 expression both in the MG-63 cells. (d) Ectopic expression of DARS-AS1 promoted cell invasion in the MG-63 cell. (e) The relative invasive cells were shown. ^∗^*p* < 0.05, ^∗∗^*p* < 0.01, and ^∗∗∗^*p* < 0.001.

CCK-8 assay results indicated that overexpression of DARS-AS1 enhanced cell proliferation in the MG-63 cells (Figure 5(a)). Ectopic expression of DARS-AS1 increased ki-67 expression in the MG-63 cells (Figure 5(b)). Elevated expression of DARS-AS1 promoted cyclin D1 expression in the MG-63 cells (Figure 5(c)). Moreover, ectopic expression of DARS-AS1 promoted cell invasion in the MG-63 cell (Figures 5(d) and 5(e)).

### 3.6. Downregulated Expression of DARS-AS1 Inhibited Cell Growth in MG-63 Osteosarcoma Cell

**Figure 6:**
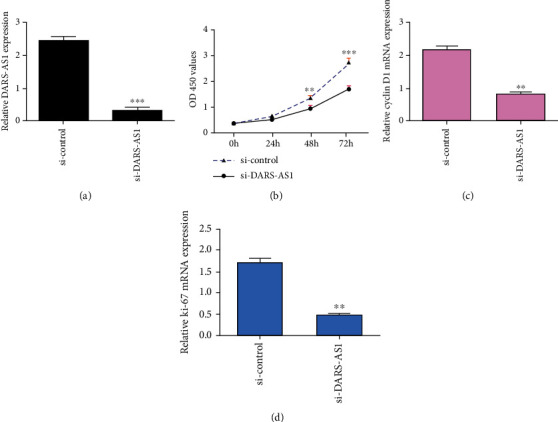
DARS-AS1 promoted cell growth in MG-63 osteosarcoma cell. (a) The expression of DARS-AS1 was detected by RT-qPCR assay. (b) Knockdown of DARS-AS1 suppressed cell proliferation in the MG-63 cells. (c) The expression of cyclin D1 was detected by RT-qPCR assay. (d) Inhibition expression of DARS-AS1 suppressed ki-67 expression. ^∗^*p* < 0.05, ^∗∗^*p* < 0.01, and ^∗∗∗^*p* < 0.001.

The expression of DARS-AS1 was significantly downregulated in MG-63 osteosarcoma cell after treated with si-DARS-AS1 (Figure 6(a)). Knockdown of DARS-AS1 suppressed cell proliferation in MG-63 cells (Figure 6(b)). Inhibited expression of DARS-AS1 suppressed the expression of cyclin D1 (Figure 6(c)) and ki-67 (Figure 6(d).

### 3.7. Elevated Expression of DARS-AS1 Enhanced Cell Growth and Invasion via Regulating CCR7

**Figure 7:**
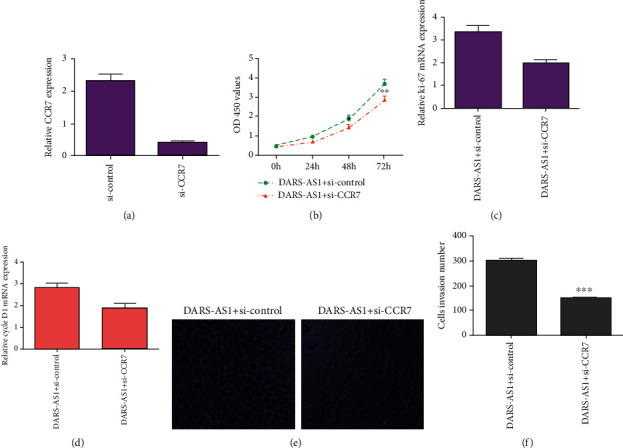
Elevated expression of DARS-AS1 enhanced cell growth and invasion via regulated CCR7. (a) The expression of CCR7 was downregulated in the MG-63 cell after treated with si-CCR7 by using qRT-PCR. (b) CCK-8 assay indicated that downregulation expression of CCR7 suppressed cell proliferation in the DARS-AS1-overexpressing MG-63 cell. (c) The expression of ki-67 was analyzed by qRT-PCR assay. (d) The expression of cyclin D1 was analyzed by qRT-PCR assay. (e) Downregulation expression of CCR7 inhibited cell invasion in the DARS-AS1-overexpressing MG-63 cell. (f) The relative invasive cells were shown. ^∗^*p* < 0.05, ^∗∗^*p* < 0.01, and ^∗∗∗^*p* < 0.001.

To study whether DARS-AS1/CCR7 derived osteosarcoma progression, rescue experiments were performed. We confirmed that the expression of CCR7 was downregulated in the MG-63 cell after treated with si-CCR7 (Figure 7(a)). CCK-8 assay indicated that downregulation of CCR7 suppressed cell proliferation in the DARS-AS1-overexpressing MG-63 cell (Figure 7(b)). Knockdown of CCR7 inhibited ki-67 (Figure 7(c)) and cyclin D1 (Figure 7(d)) expression in the DARS-AS1-overexpressing MG-63 cell. CCR7 knockdown suppressed cell invasion in the DARS-AS1-overexpressing MG-63 cell (Figures 7(e) and 7(f)).

### 3.8. Discussion

Our study identified that DARS-AS1 acted as one oncogenic lncRNA in the development of osteosarcoma. We firstly revealed that DARS-AS1 expression was higher in osteosarcoma specimens than in paired nontumor samples. DARS-AS1 promoted cell growth and invasion in MG-63 osteosarcoma cell. We found that DARS-AS1 played as a sponge for miR-532-3p in osteosarcoma cell, and ectopic expression of DARS-AS1 inhibited miR-532-3p level in MG-63 cell. Furthermore, miR-532-3p expression was lower in osteosarcoma specimens than in nontumor samples and miR-532-3p expression was negatively associated with DARS-AS1 expression in osteosarcoma specimens. miR-532-3p directly regulated CCR7 expression in osteosarcoma cell. Elevated expression of DARS-AS1 enhanced cell growth and invasion via regulated CCR7. These data suggested that DARS-AS1 exerted as one oncogene in osteosarcoma partly via regulating miR-532-3p/CCR7.

Studies revealed that DARS-AS1 exerted an oncogenic role in several human tumors such as thyroid cancer, lung cancer, myeloma, and ovarian cancer [25–28]. For example, Zheng et al. [27] revealed that DARS-AS1 expression was increased in thyroid tumor specimens and was associated with poor prognosis, distant metastasis, and pathological stage, and DARS-AS1 facilitated thyroid tumor cell migration and proliferation via regulating miR-129. Liu et al. [28] found that DARS-AS1 induced nonsmall cell lung tumor progression through modulating miR-532-3p. Yan [26] showed that DARS-AS1 was upregulated via HIF-1 in myeloma. DARS-AS1 induced myeloma cell tumorigenesis and survival via binding RBM39. Huang et al. [25] indicated that expression of DARS-AS1 was upregulated in ovarian tumor specimens, and silenced DARS-AS1 expression suppressed cell invasion, migration, and proliferation via modulating miR-532-3p. Its role in osteosarcoma remains poorly explored. We firstly studied the expression level of DARS-AS1 in osteosarcoma specimens and paired nontumor samples. Our data indicated that DARS-AS1 expression was upregulated in 73.5% (25/34) of cases with osteosarcoma. Moreover, DARS-AS1 expression was higher in osteosarcoma specimens than in paired nontumor samples. DARS-AS1 promoted cell growth and invasion in MG-63 osteosarcoma cell. These results suggested that DARS-AS1 acted as one oncogenic role in the development of osteosarcoma.

Recent references have suggested that lncRNAs played roles in a lot of tumors via modulating miRNAs expression [16, 29–31]. For instance, lncRNA HOXA-AS2 suppressed osteosarcoma cell invasion, viability, and migration via sponging miR-124-3p [32]. Li et al. [33] indicated that lncRNA NR2F1-AS1 promoted osteosarcoma progression through sponging miR-483-3p. lncRNA SND1-IT1 promoted osteosarcoma migration and proliferation through regulating miRNA-665 expression [34]. lncRNA SPRY4-IT1 induced osteosarcoma progression through sponging miR-101 [35]. Moreover, lncRNA DARS-AS1 promoted ovarian cancer cell metastasis and growth via sponging miR-532-3p [25]. We also observed that DARS-AS1 has potential binding sites of miR-532-3p in osteosarcoma. Previous study demonstrated that miR-532-3p expression was downregulated in the osteosarcoma tissues [36]. We also found that miR-532-3p expression was lower in osteosarcoma specimens than in paired nontumor samples. Moreover, the data of Pearson's correlation assay indicated that miR-532-3p expression was negatively associated with DARS-AS1 expression in osteosarcoma specimens. miR-532-3p directly regulated CCR7 expression in osteosarcoma cell. Previous study demonstrated that miR-532-3p inhibited TSCC progression through regulating CCR7 and it suggested that CCR7 might play important roles in the development of osteosarcoma [37]. CCR7 are involved in tumor migration and metastasis [38]. We firstly showed that elevated expression of DARS-AS1 enhanced cell growth and invasion via regulating CCR7. It suggested that DARS-AS1/CCR7 axis might be one novel therapeutic target for osteosarcoma.

## 4. Conclusions

Our findings showed that DARS-AS1 expression was upregulated in osteosarcoma cells and tissues, and elevated expression of DARS-AS1 enhanced cell growth and invasion via regulating miR-532-3p/CCR7. These data suggested that DARS-AS1 exerted as one oncogene in osteosarcoma partly via regulating miR-532-3p/CCR7.

## Data Availability

The authors can make data available on request through an email to the corresponding author, enrxiaoping@126.com, Prof. Dr. Ren.

## Abbreviations

miR-532-3p: microRNA-532-3p
lncRNAs: Long noncoding RNAs
ncRNAs: Noncoding RNAs
ATCC: American type culture collection
FBS: Fetal bovine serum
cDNAs: Complementary DNAs
CCR7: C-C chemokine receptor 7.
